# The Antioxidant and Hepatoprotective Potential of Berberine and Silymarin on Acetaminophen Induced Toxicity in *Cyprinus carpio* L.

**DOI:** 10.3390/ani14030373

**Published:** 2024-01-24

**Authors:** Lăcrămioara Grădinariu, Lorena Dediu, Mirela Crețu, Iulia Rodica Grecu, Angelica Docan, Daniela Ionela Istrati, Floricel Maricel Dima, Maria Desimira Stroe, Camelia Vizireanu

**Affiliations:** 1 Faculty of Food Science and Engineering, “Dunărea de Jos” University of Galați, 47 Domnească Street, 800008 Galați, Romaniamirela.cretu@ugal.ro (M.C.); iulia.grecu@ugal.ro (I.R.G.); angelica.docan@ugal.ro (A.D.); daniela.istrati@ugal.ro (D.I.I.); camelia.vizireanu@ugal.ro (C.V.); 2 Institute of Research and Development for Aquatic Ecology, Fishing and Aquaculture, 54 Portului Street, 800211 Galați, Romania; dimafloricel@yahoo.com (F.M.D.); sdesimira.icdeapa@gmail.com (M.D.S.); 3 Faculty of Engineering and Agronomy, 29 Calea Calărașilor Street, 810017 Brăila, Romania

**Keywords:** carp, nutraceutical synergy, liver health, hepatotoxicity, paracetamol

## Abstract

**Simple Summary:**

This paper investigates the antioxidant and hepatoprotective potential of berberine and silymarin against acetaminophen-induced damage in common carp (*Cyprinus carpio* L.). The findings provide insights regarding the potential use of berberine and silymarin as therapeutic agents against hepatotoxicity in fish, with implications for understanding the protective mechanisms of these substances in the context of environmental and pharmaceutical-induced stress on aquatic organisms.

**Abstract:**

Berberine (BBR) and silymarin (SM) are natural compounds extracted from plants known for their antioxidant and chemoprotective effects on the liver. The present study aimed to investigate the beneficial properties of BBR and SM and the association of BBR with SM on liver function using fish as “in vivo” models. Moreover, the study investigated their hepatoprotective role after acetaminophen (APAP) exposure. For this purpose, the fish (N = 360; 118.4 ± 11.09 g) were fed with control or experimental diets for 9 weeks. In the experimental diets, the feed was supplemented with either SM (1 g/kg feed), BBR (100 and 200 mg/kg feed), or a combination of BBR with SM (SM 1 g/kg feed + BBR 100 mg/kg feed and, respectively, SM 1 g/kg feed + BBR 200 mg/kg feed). After the feeding trial, seven fish from each tank were randomly selected and exposed to a single APAP dose. The selected serum biochemical markers, oxidative stress markers, and lysozyme activity were used to evaluate the efficiency of the supplements on carp’s health profile, particularly regarding the hepatopancreas function. Our results showed that the inclusion of SM and BBR (either as a single or in combination) reduced the serum contents of total cholesterol, triglyceride, and alanine transaminase. An increase in the high-density cholesterol was observed after the administration of BBR or BBR in association with SM. Both supplements showed hepatoprotective activity against APAP-induced hepatotoxicity, especially BBR. The ameliorative effects of SM (1 g) in association with BBR (100 mg) were highlighted by the modulation of the nonspecific immune system and oxidative stress alleviation after APAP exposure.

## 1. Introduction

In recent years, global farmed fish production has experienced significant growth due to increased seafood demand. This is driven by the consideration that fish meat consumption is a healthier alternative to other types of meat. With a total production of 87.5 million tons of aquatic animals [1], aquaculture has become an important sector in the global food industry.

However, the intensification of aquaculture is often associated with increased stocking densities and confined spaces that may contribute to the occurrence of diseases and health issues in farmed fish [2,3]. In some cases, this situation demands therapeutic treatments that are aggressive to fish. Besides that, in open production systems such as cage farms located in natural water bodies, there is a high risk of fish exposure to various environmental contaminants, including pharmaceutical residues that could concentrate in their plasma [4].

Pharmaceutical residues, including emerging compounds such as painkillers, antibiotics, cytostatic drugs, hormones, anticonvulsants, and antihistamines, can enter aquatic environments through multiple pathways. These pathways include wastewater discharges from human populations, agricultural runoff, and the release of pharmaceuticals from aquaculture operations themselves [5].

Certain pharmaceuticals, such as antibiotics, antiparasitic drugs, and some non-steroidal anti-inflammatory drugs (NSAIDs), have been associated with hepatotoxic effects in fish [6,7,8]. These compounds can rapidly reach fish through contaminated water.

The liver plays a vital role in detoxification processes, including metabolizing and eliminating foreign substances from the body [9]. Exposure to hepatotoxic pharmaceutical residues can disrupt liver function and induce liver damage in fish [2]. Pharmaceutical residues can cause, among other effects, cellular damage [10], inflammation [11], oxidative stress of liver cells and different tissues [12], activity alteration of the liver enzymes involved in detoxification and metabolic processes [13], or disturbance of intestinal microbiota [14]. All these effects can interfere with important liver functions, such as protein synthesis, lipid metabolism, and detoxification processes [15]. This disruption can have systemic effects on fish health and overall metabolism [16].

Acetaminophen (also known as paracetamol or N-acetyl-para-aminophenol; APAP) is commonly used as a pain reliever and fever reducer in humans, and it can enter aquatic environments through wastewater effluents and improper disposal [17]. Fish can come into contact with APAP through two primary pathways: waterborne exposure (bioconcentration) and dietary intake. This exposure can lead to detrimental impacts on the structure and function of fish liver [2,10,18], affecting also other aquatic organisms such as invertebrates [19]. Exposure of fish to APAP has been demonstrated to impact the activity of liver enzymes, including glutathione S-transferase (GST) and catalase. These enzymes play crucial roles in detoxification and antioxidant defense mechanisms [18]. Changes in the activities of these enzymes can compromise the liver’s ability to neutralize and eliminate toxins, leading to liver dysfunction [9,10]. Histological studies have revealed pathological changes in the liver of fish exposed to APAP, including cellular damage, vacuolation, and alterations in liver architecture [20,21]. In fish, APAP induces hepatotoxicity and oxidative stress under chronic or acute exposure, to relevant environmental concentrations [2,18], respectively, to a high single dose (e.g., 500 mg/kg body weight) [21]. Therefore, APAP was selected as the drug to challenge the fish after long-time administration of SM and BBR (as single supplements or nutraceutical combinations) in the carp feed.

In the general context of climate change, the adverse impact of pharmaceutical residues on fish is expected to intensify [22]. Therefore, it is crucial to identify methods for reducing the risk of exposure of aquatic animals contaminants or finding nutritional solutions to improve aquatic animals’ health and achieve better quality of aquatic feed in the aquaculture sector. Recently, herbal plants have been incorporated into aquafeeds due to their capacity and favorable attributes that enhance growth, immunological and antioxidative responses, and resilience to different diseases [23]. They also help alleviate the negative effects of exposure to aquatic pollutants in various fish species [24]. Silymarin is a natural compound extracted from the milk thistle plant (*Silybum marianum* L.) comprising, principally, active molecules, such as flavonolignans (isosilibinin A and B, silibinin A and B, silydianin, and silychristin) or small amounts of flavonoids [25]. Known especially for its antioxidant and chemoprotective effects on the liver [26,27,28], SM is also studied for its protective and regenerative properties for other tissue, such as nervous, renal, and cardiac muscle tissues [29].

Regarding its use in fish farming, some studies investigated the potential effects of silymarin on fish growth, health, and disease prevention. The results showed that dietary supplementation with silymarin improved the growth performance and health status of fish in several species (e.g., *Pangasianodon hypophthalmus* [30], *Scophthalmus maximus* [31], *Oreochromis niloticus* [32], or *Oncorhynchus mykiss* [26], *Paralichthys olivaceus* [33], *Dicentrarchus labrax* [34], or *Cyprinus carpio* [35]).

Berberine has been used in traditional medicine for its beneficial effects on liver health. Numerous studies have investigated the hepatoprotective effects of BBR in both animal models and human subjects [36]. In fish, BBR has been previously tested, especially for its capacity to alleviate stress induced by a high-fat or high-carbohydrate diet.

In fish species like Siberian sturgeon (*Acipenser baeri*) [37] or Nile tilapia (*Oreochromis niloticus*) [38], BBR acted as an appetizer and growth promotor. BBR has also been found to exhibit anti-inflammatory and anti-fibrotic properties, contributing to its hepatoprotective actions [39,40]. Moreover, BBR can protect liver cells from damage by scavenging free radicals and inhibiting oxidative stress [41]. Berberine suppressed inflammation in the liver of yellow drum (*Nibea albiflora*) fed a soybean-oil-based diet by inhibiting pro-inflammatory cytokines and signaling pathways, preventing further injury [42].

Overall, these studies suggest that both SM and BBR may have potential benefits for the growth and health of different fish aquaculture species. Nevertheless, there are no available studies using the combination of BBR and SM in fish feed. 

Although the threats related to pharmaceutical residues in surface waters are real, as they can affect fish health, studies investigating the potential of supplements such as BBR or SM to counteract their effects are scarce. Thus, it has been demonstrated that SM reduced negative effects induced by a sublethal level of deltamethrin in common carp [43] and ameliorated the lesions caused by oxidative stress and hepatotoxicity in common carp exposed to sublethal cadmium chloride toxicity [44] or in African catfish (*Clarias gariepinus*) subjected to fluoride toxicity [45]. Likewise, BBR has demonstrated protective effects in chronic copper-induced liver and gill injury in freshwater grouper (*Acrossocheilus fasciatus*), through enhanced antioxidant capacity, reduced inflammation responses, and mitigation of the liver and gill’s structure damage [46]. Starting from the premise that both compounds have, in general, detoxifying potential and that some medical studies on mice highlighted the potential of SM [27,47,48] or BBR [49,50] to protect the liver against acetaminophen-induced damage, we directed the current study to evaluate these nutraceuticals in mitigating APAP toxicity in carp. 

In summary, this research seeks to assess, through hemato-biochemical, immunological, and antioxidant responses, the effects of BBR, SM, and their synergistic activity to enhance the growth performance and health status of *Cyprinus carpio* fingerlings (1) and to evaluate their potential to mitigate the toxic effects induced by APAP (2).

## 2. Materials and Methods

### 2.1. Experimental Design and Fish Maintenance

All experiments and animal care procedures were approved by the Ethics Committee of the “Dunărea de Jos” University of Galați (Experimental Certificate of Animal Use No. 783/2022). The experimental fish (common carp fingerlings) were purchased from a local farm and transported to the Romanian Center for Modeling of Recirculating Aquaculture Systems, facilities at “Dunărea de Jos” University of Galați. The RAS was previously described by Dediu et al. [51]. All fish were managed following the guidelines outlined in the EC Directive 86/609/EEC [52], which addresses to the welfare of animals used for experimental and scientific purposes.

**Figure 1:**
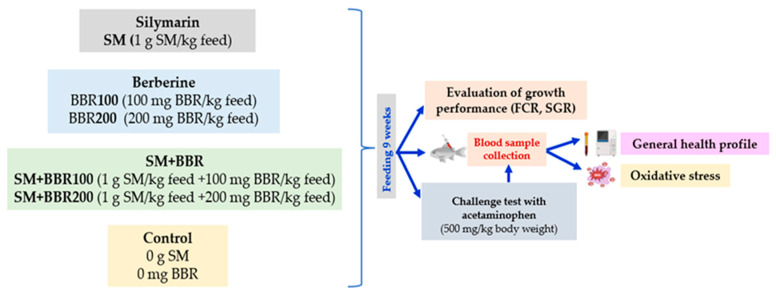
Experimental design.

Fish were acclimatized in RAS conditions for 2 weeks; during the acclimation period, no mortality occurred. After this period, a total number of 360 fish, common carp, *Cyprinus carpio*, with mean body weight of 118.40 ± 11.09 g/fish were stocked in 18 fiberglass tanks (water volume 500 L) to create six experimental groups with triplicates for each group: M (control group fed with normal diet), SM (fish fed with a diet supplemented with SM 1 g/kg feed), BBR100 (fish fed with diet supplemented with BBR 100 mg/kg feed), BBR200 (fish fed with a diet supplemented with BBR 200 mg/kg feed), SM + BBR100 (fish fed with a diet supplemented with SM 1 g/kg feed and BBR 100 mg/kg feed), and SM + BBR200 (fish fed with a diet supplemented with SM 1 g/kg feed and BBR 200 mg/kg feed) (Figure 1). The photoperiod was 16 h of light and 8 h of darkness. The commercial diet had a content of 40% protein, 10% fat, 1.5% fiber, and 6.8% ash. (Skretting, Mozzecane, Italy). The fish were fed to apparent satiation twice per day for 9 weeks.

Silymarin and berberine were purchased from Swanson Health Products (SHP), (Swanson Health Products, Fargo, ND, USA). To incorporate the specified supplement into the meal, the basal diet was initially ground and then combined with the designated supplements, processed into pellets, and dried. Following the drying process, the pellets were transferred into plastic bags and subsequently stored in a freezer at a temperature of −20 °C until they were ready for feeding. 

After 9 weeks, 7 fish from each tank were randomly selected and retained for a “challenge test” consisting in a dose of 500 mg/kg body weight of acetaminophen administered orally via gavage. The test was conducted to assess whether the supplements ameliorate the deleterious effects caused by APAP in carp. The “challenge test” was performed in 18 glass aquariums, each with a volume of 130 L.

### 2.2. Water Quality Parameters

During the experimental period, daily monitoring of water quality parameters was conducted. The Endress+Hauser monitoring system (Endress+Hauser AG, Reinach, Switzerland) was employed to automatically measure water temperature, dissolved oxygen, and pH. Probes were placed in each tank for this purpose. Additionally, the concentrations of nitrogen compounds were quantified weekly using a Skalar SAN++ analyzer from Skalar Analytical, Breda, The Netherlands. Therefore, during the experiment, the water quality parameters were monitored daily. The average values maintained within the optimal range for carp growth [53]: temperature 21.8 ± 3.14 °C; pH = 7.41 ± 1.04; dissolved oxygen 7.29 ± 1.13 mg/L; ammonium 0.09 ± 0.05 mg/L; nitrates < 0.16 mg/L; and nitrite < 0.09 mg/L.

### 2.3. Growth Performance and Organosomatic Indices

At the end of the trial, all fish were weight and measured. Growth performance and feed utilization parameters were calculated according to the following equations:

- Weight gain (WG, g) = final body weight (g) − initial body weight (g);
- Specific growth rate (SGR, %/day) = 100 × [Ln (final body weight) − Ln (initial body weight)]/days;
- Feed conversion ratio (FCR) = Consumed feed (g)/WG (g).

To calculate organosomatic indices, at the end of the experiment fish were sacrificed and the weight of the liver, gonads, and spleen were assessed.

- Hepatosomatic index (HSI, %) = [liver weight (g)/body weight (g)] × 100;
- Gonadosomatic index (GSI, %) = [gonads weight (g)/body weight (g)] × 100;
- Spleen somatic index (SSI, %) = [spleen weight (g)/body weight (g)] × 100.

### 2.4. Biochemical Composition of Carp Muscle

The biochemical determinations were performed on muscle tissue samples, following the procedures outlined by the Association of Official Analytical Chemists [54]. The ash content (%) of the sample was determined using a muffle furnace (Nabertherm, Lilienthal, Germany, Applied Scientific Instruments Co., Ltd., Bangkok, Thailand) at 525 ± 25 °C for 8 h. The moisture content (% dry weight) was determined by drying the meat samples at 105 °C in a convection oven (Jeiotech, Jeio Tech Co., Inc., Seoul, Republic of Korea) until a constant weight was obtained. After determining the moisture content, dry samples were finely ground and used to determine of protein and fats. Crude protein content (%) was calculated by converting the nitrogen content (using the common conversion factor of N × 6.25), quantified by Dumas’s method, through combustion of dry samples at 1100 °C (Primacs SNC 100, Skalar Analytical B.V., Breda, The Netherlands). Lipid content (%) in fish tissue was analyzed using the Soxhlet extraction method with petroleum ether as the solvent (C. Gerhardt GmbH & Co. KG, Königswinter, Germany). 

### 2.5. Hematological Analysis

Hematological, biochemical, and antioxidant assays were conducted on blood samples collected from ten fish per treatment at the end of the 9-week feeding trial and after the administration of APAP dose. Before blood collection, all fish were anesthetized in water with 0.3 mL/L of 2-phenoxyethanol, until reaching a state of deep anesthesia [55].

Blood was sampled from the fish caudal vein using heparinized syringes and transferred to Eppendorf sterilized tubes. The process was conducted under chilled conditions until the samples were treated in the laboratory for additional analysis. Plasma was acquired through blood centrifugation at 3500 rpm (1166× *g*) for 10 min, and it was subsequently utilized for additional biochemical analyses.

Hematological parameters such as red blood cell count (RBC × 10^6^/µL) were assessed following the method described by Svoboda et al. [56], using a Neubauer hemocytometer, glass blood diluting pipette and Vulpian diluting solution, prepared in the laboratory from sodium citrate, potassium iodide, and metallic iodine (Sigma-Aldrich, St. Louis, MO, USA). 

Hemoglobin (Hb, g/dL) levels were determined using the cyanmethemoglobin method [57] with Drabkin’s reagent (DIALAB, Wiener Neudorf, Austria). The absorbance was subsequently measured at a wavelength of 540 nm using a Specord 210 UV–Vis spectrophotometer (Analytic Jena, Jena, Germany).

For hematocrit measurement (Ht, %), 30 µL of heparinized blood was transferred into hematocrit microcapillary tubes. After that, the tubes were centrifuged for 5 min at 12,000 rpm (13,709× *g*) with the centrifuge (Hettich Haematokrit 210, Andreas Hettich GmbH & Co. KG, Tuttlingen, Germany). The values were evaluated by using the centrifuge’s integrated measuring lid. 

Also, the other hematological indices like mean corpuscular volume (MCV, fL), mean corpuscular hemoglobin (MCH, pg), and mean corpuscular hemoglobin concentration (MCHC, g/dL) were calculated from the Ht, Hb, and RBC [58,59].

### 2.6. Analysis of Blood Biochemistry

For the biochemical analyses, we used the VetTest^®^ Chemistry Analyzer and IDEXX VetTest kits (IDEXX Laboratories, Inc., Westbrook, ME, USA) for triglyceride (TG, mg/dL), cholesterol (CHOL, mg/dL), low-density lipoprotein (LDL, mg/L), high-density lipoprotein (HDL, mg/dL), alanine amino-transferase (ALT, U/L), aspartate amino-transferase (AST, U/L), alkaline phosphatase (ALP, U/L), gamma glutamyl-transferase (GGT, U/L), direct bilirubin (BILD, mg/dL), and total bilirubin (BILT, mg/dL). 

### 2.7. Analysis of Oxidative Stress, Lyzozyme Activity, and Total Antioxidant Capacity

Lipid peroxidation concentrations were quantified using the Ohkawa method [60] by reading the optical density of samples at 532 nm. The results were reported as nmol of malondialdehyde (MDA) per mL of plasma or gram of liver homogenate (nmol/g liver).

Lysozyme activity (LYZ, U/mL) was assessed using the Enzymatic Activity of Lysozyme Protocol (Sigma, EC 3.2.1.17, Sigma-Aldrich, St. Louis, MO, USA).

The total antioxidant capacity (TAC, mMol Trolox) was determined using the method described by Roberta et al. [61]. The spectrophotometric measurement was conducted at an optical density of 734 nm. 

### 2.8. Data Analysis

The statistical analyses were conducted utilizing the SPSS statistical software for Windows, Version 26.0, Chicago, IL, USA, SPSS Inc. Data examination was made using one-way ANOVA, followed by Duncan’s post hoc test for multiple group comparisons (*p* < 0.05). Before statistical analyses, normality and homogeneity of variance were ensured by performing Shapiro–Wilk and, respectively, Levene’s tests. Additionally, the differences in the means of hematological and plasma biochemical parameters at the two sampling time points (before and after the challenge test) were compared using a T-dependent test (*p* < 0.05) for each treatment diet.

## 3. Results

### 3.1. Evaluation of Growth Performance

At the beginning of the experiment, the average initial body mass of the carp specimens did not show statistically significant differences (*p* > 0.05) among the six experimental variants. By the end of the feeding trial, the mean weight of the carp registered significantly different values (*p* ˂ 0.05) among these six experimental variants (Table 1). 

The highest average body mass was observed in the BBR200 and SM experimental variants. In terms of specific growth rate (SGR), the groups fed only with SM or BBR performed similarly, with significantly higher values than the control groups (M) or the groups fed with SM + BBR association. The lowest feed conversion ratio (FCR) was also found in SM and BBR200 variants.

### 3.2. Evaluation of Organosomatic Indices and Muscle Biochemical Composition of Carp

At the end of the experiment, a significant lower HSI (*p* < 0.05) was calculated for SM, BBR100, BBR200, and SM + BBR100, while for GSI, the lowest values were observed for SM and BBR200. No significant differences (*p* > 0.05) between variants were detected for SSI (Table 2).

Regarding muscle biochemical composition, the statistical analysis did not reveal significant differences (*p* > 0.05) in terms of water, protein, and ash content. In contrast, lipid content was significantly lower (*p* ˂ 0.05) in all the variants in which the bioactive compounds were supplemented in the fish feed (Table 3).

### 3.3. Evaluation of Hematological and Biochemical Blood Parameters

At the end of the feeding trial and after the APAP challenge, fish hematological parameters were determined, and the results are presented in Table 4. Thus, after the feeding experiment, statistical analysis revealed significant differences (*p* ˂ 0.05) only in terms of RBC values, with higher values for SM + BBR200, while Ht, Hb, VEM, HEM, and CHEM values did not show significant differences (*p* > 0.05) among the six experimental variants. Similarly, after the APAP test, the values of hematological parameters did not indicate significant differences (*p* > 0.05) among the six experimental variants. However, the statistical comparison (dependent T-test) before and after the challenge test revealed a significant decrease in Ht mean values in all experimental variants, with the obtained values falling within the optimal range found in the literature (Table 5). 

### 3.4. Blood Metabolic Profile

At the end of the feeding trial and after the APAP challenge, the metabolic blood profile was analyzed for all experimental variants, and the results are presented in Table 6.

After the feeding trial, the TG, CHOL, and LDL values reduced significantly (*p* < 0.05) compared to the control variant. HDL increased significantly (*p* < 0.05) in BBR200 followed by BBR100 and SM + BBR200 and SM + BBR100. No significant differences (*p* > 0.05) were detected between all tested variants, for AST, ALP, and GGT. However, significantly lower (*p* < 0.05) ALT values are observed in all variants where BBR was supplemented in the feed. The values of tested biomarkers varied within the range considered normal for the studied species [70].

After the APAP administration, significant changes were noticed in all analyzed metabolic markers (*p* < 0.05) for the control variant (M), indicating liver toxicity reflected mainly by the hepatic enzymes released in higher amounts into the blood stream, up to 400% (i.e., ALT). Most hepatic biomarkers registered insignificant increased values (*p* > 0.05) after exposure for groups receiving BBR or a BBR + SM enriched diet.

### 3.5. Oxidative Stress Parameters and Lysozyme Activity

**Figure 2:**
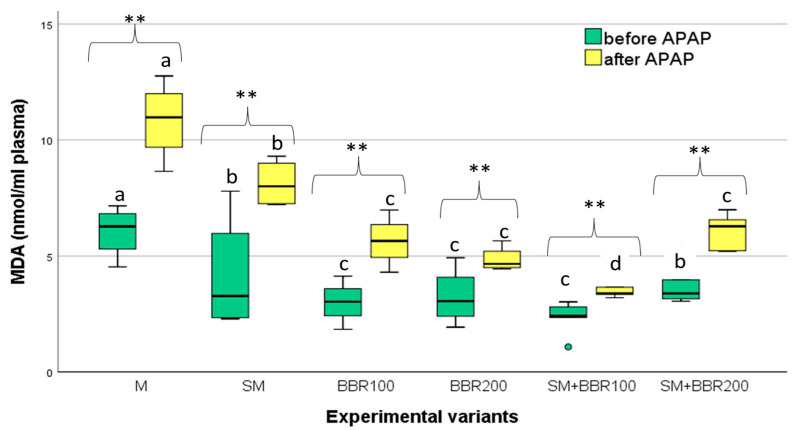
MDA Variation—Boxplot (median, minimum, maximum, and quartiles). Values with different letters indicate significant differences (ANOVA, *p* ˂ 0.05) between experimental variants. Values with ** indicate significant differences after the APAP test (T-dependent, *p* ˂ 0.05).

Oxidative stress parameters and lysozyme activity were assessed after the feeding trial, as well as after the APAP challenge test. After the feeding trial, MDA serum concentration had the lowest mean value in SM + BBR100, followed by BBR100, BBR200, SM + BBR200, SM, and M variants (Figure 2). SM and BBR administration significantly reduced serum MDA contents elevated by acetaminophen exposure (*p* < 0.05); the association of SM with 100 mg BBR reduced MDA production more effectively compared with the other treatments.

**Figure 3:**
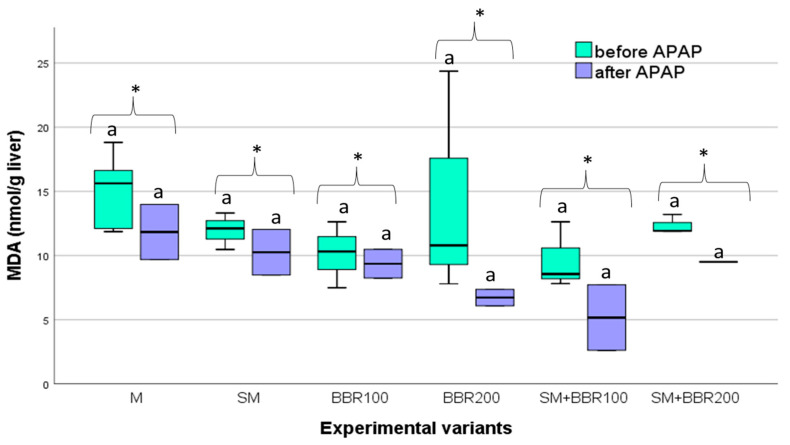
Liver MDA Variation—Boxplot (median, minimum, maximum and quartiles). Values with different letters indicate significant differences (ANOVA, *p* ˂ 0.05) between experimental variants. Values with * indicate non-significant differences (T-dependent, *p* > 0.05) after the APAP test.

Regarding the MDA values in the liver, there were no significant differences (*p* ˃ 0.05) among treatments, both after the feeding experiment and the challenge test. Moreover, the comparison of MDA values before and after the challenge test revealed insignificant differences (*p* ˃ 0.05) for all treatments (Figure 3).

**Figure 4:**
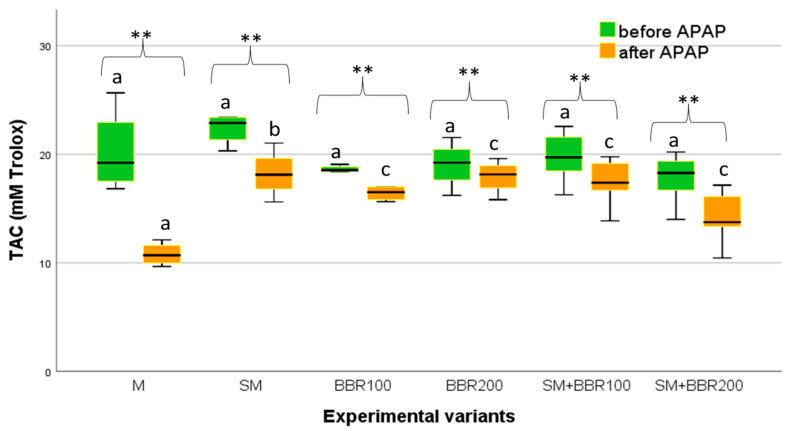
TAC Variation—Boxplot (median, minimum, maximum and quartiles). Values with different letters indicate significant differences (ANOVA, *p* ˂ 0.05) between experimental variants. Values with ** indicate significant differences after the APAP test (T-dependent, *p* ˂ 0.05).

After the feeding trial, the level of total antioxidant capacity (TAC) quantified for the plasma did not show significant differences (*p* > 0.05) between the six experimental variants (Figure 3). However, after APAP exposure, TAC had the lowest mean values in the control variant and the highest in the SM variant (Figure 4).

**Figure 5:**
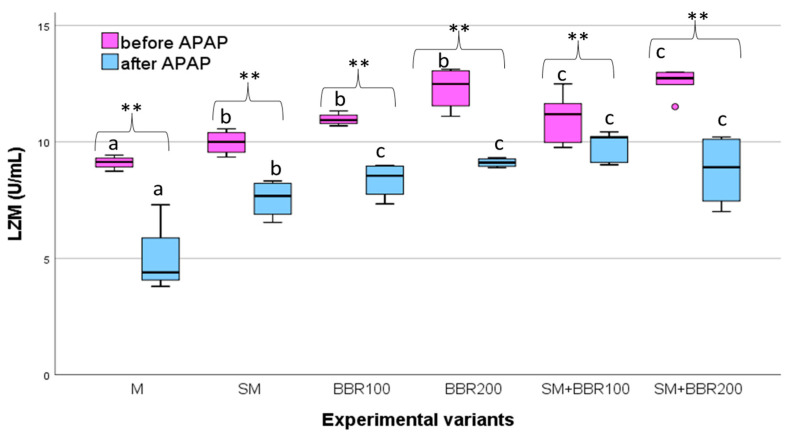
LZM Variation—Boxplot (median, minimum, maximum and quartiles). Values with different letters indicate significant differences (ANOVA, *p* ˂ 0.05) between experimental variants. Values with ** indicate significant differences after the APAP test (T-dependent, *p* ˂ 0.05).

Regarding the LZM activity, at the end of the feeding experiment, the statistical analysis revealed significant differences (*p* ˂ 0.05) between the six experimental variants, with the LZM activity from the BBR200 and SM + BBR200 variants being significantly higher compared to SM + BBR100, BBR100, and SM variants. The lowest activity of LZM was observed in the control variant (Figure 5). 

After the challenge test, individuals from the M variant had significantly lower (*p* ˂ 0.05) LZM activity, compared to experimental groups receiving BBR as the sole supplement or in combination with SM; LZM activity for groups receiving only SM was significantly lower (*p* ˂ 0.05) than other groups receiving BBR in the diet.

## 4. Discussion

The use of plants or plant extracts in fish feed has become a widely accepted practice due to their benefits in enhancing fish growth, improving immunity, and increasing survival rates [71,72,73]. Although there are numerous reports regarding the immunostimulant properties of plants and plant extracts (including algae) and the utilization of plant additives for pathogen control [74], data related to their protective role against the toxicity of different compounds are still limited.

Among other plants or plant extracts, SM and BBR have been used in aquaculture as functional feed additives to enhance growth, improve immune response and antioxidative capacity, or increase disease resistance of the fish [26,30,32,38,39,40,75,76,77]. However, there is limited information regarding the supplementation of carp feed with SM and BBR, and no information regarding their combination. The present study has assessed the impacts of BBR-, SM-, and SM + BBR-supplemented diets, after 9 weeks of feeding, on the overall growth performance and health profile of the *C. carpio* juveniles and investigated the alleviation of APAP-induced toxic effects. To achieve this objective, growth performance, serum biochemical variables, immune and antioxidant biomarkers have been assessed.

After 9 feeding weeks, the results highlighted that SGR values were significantly higher in the groups where SM and BBR were administered as sole supplements, while the combination of SM with BBR did not result in superior growth performance compared with the control variant. In other studies, including SM in the diet of aquatic organisms led to different results depending on the species, the SM concentration, or the feed formulation. Thus, SM improved the growth performance of turbot (*Scophthalmus maximus)*, when the plant protein diet was supplemented with 100 mg/kg [31], and of grass carp juvenile (*Ctenopharyngodon idella*) at a 60 mg/kg feed concentration [78]. In a similar trial, it was found that SM at 100–200 mg/kg can significantly improve the growth performance and health status of juvenile grass carp, with better results for 100 mg/kg than 200 mg/kg [79]. On the other hand, in contrast with our results, in a trial on common carp (*Cyprinus carpio*) the growth parameters including final weight, final weight gain, FCR, and survival rate showed no significant difference between all groups after feeding SM in concentrations ranging from 400 to 2400 mg SM/kg diet [44], while for rainbow trout (*Oncorhynchus mykiss*), the oral administration of 800 mg SM/kg feed caused cytotoxicity and modifications in blood biochemical parameters of the fish [26]. 

Historically, BBR has also been investigated for its potential to address metabolic disorders linked to high-lipid or high-carbohydrate diets in fish. In recent years, the positive effects of berberine on the productive performance of fish have been highlighted in several studies. In our study, the administration of BBR in carp diet resulted in superior SGR, for both concentrations (100 and 200 mg/kg feed), compared with the control variants; nevertheless, better feeding efficiency was observed in groups fed with higher BBR concentration (in BBR200). A comparable improvement in growth performance was documented for black carp (*Mylopharyngodon piceus*), which had a superior growth performance after administration of BBR (98.26 mg/kg and 196.21 mg/kg) in feeds with a high lipid content [80], for largemouth bass (*Micropterus salmoides*) provided with 1 g/kg berberine [81] or for Nile tilapia (*Oreochromis niloticus)* receiving up to 9 g/kg berberine as supplements in normal diets [38]. However, an excessive intake of dietary BBR has been observed to impede fish growth, as evidenced in black carp fed with 392.07 mg/kg berberine [80] or blunt snout bream (*Megalobrama amblycephala*) fed with 100 mg/kg berberine [75]. In a similar study, the supplementation of high-lipid feed with 50 mg BBR/kg was conducted to improve growth performance and feed consumption for blunt snout bream, while administration of 100 mg BBR/kg led to the rejection of the feed by the fish and, respectively, to poorer growth performance, the result being attributed to the bitter taste of BBR [75]. High variable results reported in the literature suggest that the growth-promoting effect of BBR varies greatly among species. Some authors have reported that the long-term use of BBR in fish feed may even reduce its beneficial effect [82]. This effect has been reported inclusively for mammals, with the hypothesis being issued that intestinal expression of P-glycoprotein is overwhelmed by high doses or long-term administration of BBR, which inhibits the intestinal absorption of berberine [80]. However, this mechanism has not been reported in fish and should be further studied. Across fish species, feeding methods, basal diet compositions, and other unidentified factors also affected the variability of the results.

Regarding organosomatic indices, in the present experiment, significant differences were detected among experimental variants. Thus, compared with the control, a decrease in the HSI index can be observed in the variants in which the feed was supplemented with bioactive ingredients, except for the SM + BBR200 variant. A lower HSI indicates a reduction in fat accumulation in the liver [42], suggesting that SM and BBR had a beneficial effect on the liver health of the fish. It is known that fatty liver can lead to metabolic pathologies in mammals, from steatosis to hepatocellular damage, fibrosis, or liver failure [83]. On the other hand, fish are more susceptible to fat accumulation in the liver because the liver is the main storage location for lipids. In recent years, in aquaculture, increasing deficiencies and a high mortality rate caused by fatty liver have been reported, causing significant economic consequences [84].

In general, the biochemical composition of the meat reflects the quality of the fish and is influenced by several factors, such as feed composition and feeding method [85]. Although the protein values did not register significant differences among the experimental variants, a slight increase in the protein percentage can be observed in the variants in which food additives were used in the carp feed; a significant reduction in lipids was also observed in these variants. Thus, these results suggest that food additives can be effectively used to improve the quality of fish meat and satisfy the nutritional requirements of the consumers.

The general health profile of the fish is assessed through various instruments, with hematological parameters occupying an important position [26]. In our study, the analysis of hematological parameters did not reveal major changes, after the administration of the selected food supplements, except for RBC, which significantly increased in the SM + BBR200 variant, registering the highest value. The administration of SM alone slightly decreased RBC, Ht, and Hb, while in combination with BBR, it increased Hb and RBC. However, the comparison of mean values did not show statistical differences among treatments, and the parameters were within the range of values considered normal for the species under study. Our results are thus only partially supported by similar studies. In rainbow trout, after the administration of SM (0.1, 0.4, and 0.8 g/kg food), a significant increase in the number of erythrocytes, hematocrit, and hemoglobin concentration was observed, highlighting the influence of SM on hematopoietic organs, such as the spleen and kidney, which play an essential role in the formation of blood cells [86]. The administration of 500 mg/kg of barberry root extract significantly increased WBC, RBC, Hb, and MCHC in rainbow trout [87] and increased MCV and Ht in yellow catfish, *Pelteobagrus fulvidraco*, fed with a diet containing 400 mg berberine/kg for 60 days [88].

Regarding the influence of APAP on hematological parameters, a significant decrease in Ht was observed in all experimental variants after the challenge test. Moreover, an increase in RBC and a decrease in MCV were registered in the control variant, while for the SM + BBR200 experimental variant, a significant decrease in RBC was observed. This aspect could suggest different mechanisms of the blood system of fish fed with different diets in order to cope with the toxic agent. 

Plants and plant extracts like SM or BBR often possess natural compounds that can help mitigate the harmful effects of toxic compounds in fish. Thus, it has already been demonstrated that SM or BBR has protective effects against different toxic compounds like cadmium chloride [44], paraquat [89], and deltamethrin [43] in fish species. In this context, it was hypothesized that SM and BBR could have stronger protection against the toxicity of pharmaceutical drugs when administrated in a combination. 

APAP is often selected as a hepatotoxicant for inducing liver injury, as it is known to cause hepatotoxicity when taken in overdose, leading to the elevation of liver enzymes in experimental animals [90,91] including fish [92]. 

AST is an enzyme found in the mitochondria and cytoplasm of all cells, while ALT is a hepatocellular cytoplasmic enzyme whose elevation in blood indicates liver damage. Elevated levels of AST and ALT in the blood may indicate cellular leakage and loss of functional integrity of the hepatocyte cell membrane induced by any toxic substance [93]. Elevated serum ALP levels can indicate kidney and liver damage [94] and increased biliary pressure [95].

According to our findings, after the administration of SM and BBR doses, no significant differences were noticed between the tested variants regarding the level of AST and ALT enzymes in the blood; these levels fell within the range of values considered normal for carp [70]. However, the administration of BBR in both concentrations (100 and 200 mg/kg) resulted in lower ALT values, suggesting an improved liver function. After APAP administration, the levels of plasma liver enzymes (ALT, AST, and ALP) increased significantly for the control, emphasizing liver dysfunction. ALT values for SM and all BBR treatments increased insignificantly (*p* > 0.05), while AST values had an insignificant increase in SM and BBR + SM treatments in comparison with the control. These results indicate a reduction in ALT and AST enzyme activities for SM, SM + BBR100, and SM + BBR200 treatments, suggesting an alleviation of APAP detrimental effects for these treatments.

Oral SM in other species, such as *Oncorhynchus mykiss*, stabilized cell membrane structure and regulated AST, ALT, and ALP activity levels at concentrations up to 400 mg/kg body weight [26]. A similar study, carried out on the species *Cyprinus carpio*, showed that SM protects hepatocytes against tissue damage induced by cadmium chloride only at concentrations of 2400 mg/kg diet [44].

In various laboratory animal studies, the administration of BBR has been shown to reduce hepatotoxicity induced by various pharmaceutical compounds such as methotrexate [41], metformin [96], and gentamicin [97]. The hepatoprotective effect of berberine derives from the fact that this isoquinoline alkaloid compound is biologically active with antihyperglycemic, antioxidant, anti-inflammatory, antimicrobial, anticarcinogenic, and renoprotective properties [98]. At the same time, the obtained results can also be supported by the protective effect that BBR has against free radicals, being a good scavenger for reactive nitrogen species [39].

Throughout the history of medicine, the fruits of plants such as *Berberis vulgaris* have also been used for beneficial effects on the biological function of the liver and the cardiovascular system by reducing triglycerides, cholesterol, low-density lipoproteins, and blood pressure due to its unique bioactive compounds, such as BBR [99,100].

Triglycerides (TG) are the main form in which lipids are stored to provide energy to fish [101], bringing information on lipid energy reserves. Thus, the plasma triglyceride (TG) level can be used as an indicator of nutritional status, as well as the total plasma protein and cholesterol level. In our study, plasma TG levels of the fish fed with BBR, SM, and BBR + SM enriched feed decreased significantly (*p* < 0.05) compared to the control group. The lowest TG values were found in the SM + BBR100 and SM + BBR200 variants suggesting the synergistic effect of SM and BBR. Similar results were reported for the sturgeon *Acipenser baeri* [37], whose diet, supplemented with BBR in concentrations of 150, 300, 600, and 750 mg/kg, was negatively correlated with the fish plasma TG values. BBR has also been reported to be an effective serum lipid-lowering agent by reducing TG synthesis through stimulating the activity of adenosine monophosphate (AMP)-activated protein kinase, which plays a key role in cellular energy homeostasis [102].

Regarding total cholesterol (CHOL), after the feeding experiment, the mean plasma values were significantly lower in the experimental variants in which food was enriched with SM, BBR, or both supplements. However, it should be noted that CHOL had the lowest values in the groups receiving BBR, either as a single supplement (in BBR100) or in a nutraceutical combination with SM (in SM + BBR200). Additionally, the cholesterol fraction values show that BBR positively influenced the HDL/LDL ratio; the best ratio was found for BBR200 and BBR100. Similar results, consistent with the present study, were obtained for black carp (*Mylopharyngodon piceus*) [80] and black sea bream [103], after BBR supplementation in a high-lipid diet, when reduced TG, CHOL, and LDL concentrations were registered and HDL concentration increased.

A high APAP dose rapidly depleted liver levels of triglycerides and cholesterol, indicating the impairment of carbohydrate and lipid metabolism. A loss of glycogen or lipids may occur as a direct effect of intoxication or may occur secondarily, as a result of altered general body condition caused by starvation, stress, or concomitant illness [104].

Both subtoxic and toxic doses of APAP have also been shown to downregulate genes of energy-consuming biochemical pathways, including gluconeogenesis (glucose-6-phosphatase), fatty acid synthesis (sterol-C4-methyl oxidase), and cholesterol synthesis (3-hydroxy-3-methylglutaryl-coenzyme A synthase 1) and regulate energy-producing biochemical genes such as glycolysis/gluconeogenesis (6-phosphofructo-2-kinase/fructose-2,6-bisphosphatase 1) [105]. In the case of fish fed with SM and BBR (experimental variants SM and BBR100), the level of triglycerides decreased insignificantly (*p* > 0,05) after exposure to APAP, suggesting the regulatory action of these supplements.

Cholesterol has also been used in numerous previous research studies as a diagnostic tool for biological monitoring of the condition of farmed fish [106]. Consequently, elevated energy metabolites such as CHOL, HDL, and LDL may indicate lipid or lipoprotein metabolic problems or liver dysfunction. The results obtained after the toxic challenge test revealed that the level of total cholesterol and cholesterol fractions decreased for all experimental variants. However, it can be easily noted that, from the perspective of lipid metabolism, the control variant was the most exposed to the negative effect of APAP-induced hepatotoxicity, while the variants with insignificant decreases in HDL were those fed with diet containing BBR. Also, BBR100, BBR200, and SM + BBR2000 variants recorded the least depreciation of CHOL values, while BBR200 had the least HDL depreciation.

GGT (γ-glutamyl transferase), also known as gamma-glutamyl transpeptidase, is an enzyme present mainly in the liver, but also in other tissues such as the kidneys, pancreas, and spleen. High concentrations of GGT in the blood can indicate various liver diseases, especially damage to the bile ducts. Bilirubin is a yellow-orange pigment resulting from the breakdown of hemoglobin in old erythrocytes.

In the present study, after the feeding experiment, no significant differences were found between the mean GGT and BILD values of the tested experimental groups (*p* > 0.05 in the ANOVA test). However, the BILT value, increased significantly in the control group compared to the experimental groups (*p* < 0.05). After APAP exposure, the values of GGT, BILD, and BILT increased significantly (*p* < 0.05) in the control groups, while for the experimental variants in which the bioactive compounds were tested, the mean GGT, BILD, and BILT showed an insignificant (*p* > 0.05) increase.

The ameliorative effects of BBR and SM were thus highlighted by modulating the activity of GGT and bilirubin. Bilirubin is considered one of the most potent endogenous antioxidants, and its serum concentrations are predominantly affected by hepatic bilirubin activity. Thus, bilirubin intervenes in the protection of the cells when they are exposed to the negative effects of toxic compounds [107]. High bilirubin values in the control variant indirectly reflect the impairment of liver function due to APAP-induced hepatotoxicity. At the same time, the protection provided by SM against APAP-induced liver toxicity can be generally related to several beneficial properties, including free radical scavenging, increased cellular growth somatic hormone content, and membrane permeability regulation [47]. Moreover, in the present case, combining SM with 100 mg BBR/kg feed resulted in a better status due to the additional contribution of BBR.

MDA is a commonly used indicator to assess lipid peroxidation. Lipid peroxidation is a chemical process by which lipids are exposed to oxidative factors, generating free radicals and toxic compounds that can damage the cellular structure, oxidative stress, and negative effects on the physiology of aquatic organisms [108,109]. In our study, a higher level of plasma MDA in the control variant, after the feeding trial, may indicate a slight imbalance between the generation and elimination of reactive oxygen species (ROS). The results showed that supplementing feed with BBR or a nutraceutical mixture of BBR and SM reduced oxidative damage in plasma by diminishing MDA production (the MDA had the lowest values in BBR100, BBR200, and SM + BBR100). However, the combination of SM with a higher BBR concentration did not yield better results. Similar results were also reported for yellow catfish (*Pelteobagrus fulvidraco*), fed with BBR 100 and 400 mg/kg feed [88], where MDA registered higher values in the BBR400 variant. In the present study, after the challenge test, the lowest MDA mean value was obtained in the SM + BBR100 variant, followed by BBR100 < BBR200 < SM + BBR200 < SM.

Regarding MDA values in the liver, although a slight decrease was observed in treatment groups, they did not show significant differences (*p* ˃ 0.05) either after the feeding experiment or after the APAP test. In line with our findings, BBR supplementation (50 mg/kg) reduced hepatic MDA in blunt snout bream [109] and in largemouth bass fed with BBR-supplemented high-starch diets [81]. BBR supplementation in a high starch diet of black sea bream also reduced MDA content after the ammonia challenge [110]. Similarly, a reduction in MDA level and increase in antioxidant capacity after the administration of SM (0, 0.1, 0.5, and 1 g/kg feed) in feed were also reported for carp [111]. 

In the current study, after the feeding experiment, the total antioxidant capacity at the plasma level showed no significant differences (*p* > 0.05) between the six experimental variants. However, a slight increase in TAC values can be observed in the control. After the administration of a single dose of APAP, significantly higher TAC values were noticed in the variant in which SM was administered, followed by the variants SM + BBR200, BBR100, BBR200, and SM + BBR10. 

The inverse relationship between lipid peroxidation and total antioxidant capacity, reflected by decreasing the MDA and increasing the percentage of antioxidants in the body, has also been reported in fish [112]. In our study, after the toxicity test, the concentration of plasma MDA increased, while the TAC values decreased in all groups; however, the positive effect of the supplements in alleviating oxidative stress was highlighted by the lower increase rate of MDA and, respectively, the decreased rate of TAC (especially in the SM + BBR100 group). Therefore, adding SM and BBR in carp feed can help maintain a balanced ratio between oxidants and antioxidants, thus preventing oxidative stress.

Regarding the LZM activity, after the feeding experiment, statistical analysis revealed significant differences (*p* ˂ 0.05) between the six experimental variants; the lowest activity of LZM was observed in the control variant followed by the SM variant. The increase in plasma lysozyme activity contributes to the acceleration of the phagocytosis process [111]. Phagocytosis is a process by which specialized cells, called phagocytes, engulf and destroy bacteria and other foreign particles. By improving this process, the fish’s immunity is strengthened, facilitating the elimination of pathogens and reducing the risk of infections. It was suggested that BBR could promote the innate immune system as it exhibited a strong anti-bacterial effect in both fish [76] and rats [113,114]. In our study, after the feeding trial, the highest LZM concentration was detected in the BBR200 variant. After the APAP stress test, the statistical analysis revealed significant differences (*p* ˂ 0.05) among the experimental variants emphasizing significantly lower activity of LZM in the control variant followed by SM < BBR100 < BBR200 < SM + BBR200 < SM + BBR100. Our results are aligned with similar reports for common carp [77] and other fish species, such as blunt snout bream [115] or Nile tilapia [38], in which it was emphasized that BBR administration improved nonspecific immune status (highlighted by LZM activity) of the fish. Nevertheless, the mechanism induced by BBR in modulating the nonspecific immune system is not fully understood. In the present study, the beneficial effects of SM and BBR association were highlighted; however, to understand the physiological mechanisms behind the results, further investigations should be carried out.

## 5. Conclusions

The general health profile of the fish improved when the diet was enriched with either SM, BBR, or a combination of both. BBR and SM enhanced the growth performance in carp when administered individually as supplements, whereas their combined use did not lead to superior performance. Feeding carp with specific supplements over an extended period significantly decreased hepatotoxicity following acetaminophen exposure. The addition of SM and BBR in the carp juvenile feed highlighted the positive effect of these additives in combating oxidative stress. Our research demonstrated that administering SM 1 g/kg feed in combination with BBR 100 mg/kg feed resulted in the mitigation of oxidative stress and modulation of the nonspecific immune system, providing evidence for the strengthening effect of this nutraceutical combination.

## Figures and Tables

**Table 1:** Specific growth rate (SGR) and feed conversion ratio (FCR) for carp reared in different experimental conditions.

Growth Parameters	M	SM	BBR100	BBR200	SM + BBR100	SM + BBR200
IW (g)	122.70 ± 10.25 ^a^	121.60 ± 9.12 ^a^	113.10 ± 7.16 ^a^	124.50 ± 5.2 ^a^	115.10± 6,2 ^a^	117.40 ± 4.65 ^a^
FW(g)	179.15 ± 18.61 ^a^	195.60 ± 10.12 ^b^	180.40 ± 19.65 ^a^	198.70 ± 18.16 ^b^	179.03± 17.46 ^a^	180.61 ± 10.12 ^a^
SGR (%/day)	0.69 ± 0.02 ^a^	0.74 ± 0.01 ^b^	0.73 ± 0.04 ^b^	0.73 ± 0.02 ^b^	0.69± 0.03 ^a^	0.68 ± 0.03 ^a^
FCR (g/g)	2,21 ± 0.04 ^b^	1.47 ± 0.08 ^a^	1.61 ± 0.05 ^b^	1.46 ± 0.03 ^a^	1.68± 0.02 ^b^	2.10 ± 0.05 ^c^

Note: IW—initial average weight; FW—final average weight; SGR—specific growth rate; FCR—feed conversion ratio; values represent the mean of triplicates ± SE. Values with different letters in a row indicate significant differences (ANOVA, *p* ˂ 0.05) among experimental variants.

**Table 2:** Somatic indices of *Cyprinus carpio* at the end of the experiment.

Body Indices	M	SM	BBR100	BBR200	SM + BBR100	SM + BBR200
HSI (%)	2.29 ± 0.18 ^b^	1.91 ± 0.24 ^a^	1.92 ± 0.38 ^a^	1.69 ± 0.68 ^a^	1.65 ± 0.21 ^a^	2.38 ± 0.74 ^b^
GSI (%)	5.07 ± 2.36 ^b^	3.03 ± 2.35 ^a^	4.32 ± 1.43 ^b^	2.96 ± 0.09 ^a^	5.40 ± 1.80 ^b^	7.28 ± 5.68 ^c^
SSI (%)	0.27 ± 0.07 ^a^	0.28 ± 0.06 ^a^	0.28 ± 0.01 ^a^	0.33 ± 0.19 ^a^	0.24 ± 0.06 ^a^	0.23 ± 0.08 ^a^

Note: Values represent the mean of triplicates ± SE. Values with different letters in a row indicate significant differences (ANOVA, *p* ˂ 0.05) among experimental variants.

**Table 3:** Effects of SM and BBR inclusion on *Cyprinus carpio* muscle proximate compositions.

Composition	M	SM	BBR100	BBR20	SM + BBR100	SM + BBR200
Water (%)	78.44 ± 1.26 ^a^	78.52 ± 0.64 ^a^	79.30 ± 0.22 ^a^	79.04 ± 1.32 ^a^	79.09 ± 1.32 ^a^	79.54 ± 0.26 ^a^
Protein (%)	17.06 ± 0.73 ^a^	18.63 ± 0.17 ^a^	17.54 ± 0.47 ^a^	17.94 ± 0.53 ^a^	17.69 ± 0.61 ^a^	17.42 ± 0.56 ^a^
Lipids (%)	2.44 ± 0.88 ^b^	1.44 ± 0.46 ^a^	1.39 ± 0.08 ^a^	1.32 ± 0.22 ^a^	1.68 ± 0.28 ^a^	1.31 ± 0.13 ^a^
Ash (%)	1.12 ± 0.07 ^a^	1.29 ± 0,09 ^a^	1.26 ± 0.05 ^a^	1.07 ± 0.05 ^a^	1.72 ± 0.65 ^a^	1.20 ± 0.24 ^a^

Note: Values represent the mean of triplicates ± SE. Values with different letters in a row indicate significant differences (ANOVA, *p* ˂ 0.05) among experimental variants.

**Table 4:** Hematological parameters of *C. carpio* in different treatments.

Time	Exp. Variants	Ht (%)	Hb (g/dL)	RBC (×10^6^/mm^3^)	MCV (μm^3^)	MCH (pg)	MCHC (g/dL)
Before challenge	M	43.15 ± 2.94 ^a^*	10.09 ± 3.24 ^a^*	1.28 ± 0.22 ^b^*	340.84 ± 35.37 ^a^*	82.90 ± 35.96 ^a^*	24.22 ± 4.22 ^a^*
	SM	36.62 ± 0.31 ^a^*	8.79 ± 0.49 ^a^*	1.23 ± 0.14 ^a^*	299.70 ± 32.24 ^a^*	72.10 ± 10.92 ^a^*	24.00 ± 1.42 ^a^*
	BBR100	42.14 ± 3.80 ^a^*	10.36 ± 0.38 ^a^*	1.34 ± 0.10 ^b^*	318.73 ± 53.26 ^a^*	78.13 ± 8.95 ^a^*	24.66 ± 1.38 ^a^*
	BBR200	44.36 ± 1.11 ^a^*	11.88 ± 1.18 ^a^*	1.42 ± 0.47 ^b^*	333.38 ± 98.70 ^a^*	88.13 ± 22.24 ^a^*	26.83 ± 7.42 ^a^*
	SM + BBR100	43.98 ± 3.78 ^a^*	12.70 ± 3.25 ^a^*	1.40 ± 0.08 ^b^*	315.58 ± 39.75 ^a^*	97.41 ±20.10 ^a^*	31.55 ± 8.61 ^a^*
	SM + BBR200	44.11 ± 3.63 ^a^*	12.44 ± 2.12 ^a^*	1.91± 0.39 ^c^*	240.85 ± 58.82 ^a^*	67.04 ± 14.48 ^a^*	28.17 ± 3.81 ^a^*
After challenge	M	31.86 ± 4.75 ^a^**	12.67 ± 2.33 ^a^*	1.52 ± 0.14 ^a^**	206.95 ± 37.10 ^a^**	80.95 ± 10.40 ^a^*	40.55 ± 9.76 ^a^*
	SM	28.57 ± 2.76 ^a^**	13.77 ± 4.70 ^a^*	1.31 ± 0.26 ^a^*	235.44 ± 49.96 ^a^*	105.66 ± 25.77 ^a^*	49.43 ± 7.16 ^a^**
	BBR100	24.41 ± 3.63 ^a^**	10.33 ± 5.03 ^a^*	1.44 ± 0.28 ^a^*	180.50 ± 31.01 ^a^**	84.21 ± 22.22 ^a^*	49.38 ± 5.74 ^a^**
	BBR200	28.13 ± 6.05 ^a^**	12.52 ± 0.70 ^a^*	1.30 ± 0.27 ^a^*	225.99 ± 74.15 ^a^*	91.63 ± 18.34 ^a^*	42.11 ± 8.41 ^a^*
	SM + BBR100	23.99 ± 5.88 ^a^**	12.99 ± 1.57 ^a^*	1.32 ± 0.48 ^a^*	215.07 ± 79.54 ^a^**	91.94 ± 28.43 ^a^*	48.21 ± 6.16 ^a^**
	SM + BBR200	26.75 ± 8.19 ^a^**	13.40 ± 3.37 ^a^*	1.19 ± 0.23 ^a^**	247.57 ± 94.17 ^a^*	109.65 ± 13.83 ^a^*	52.35 ± 4.66 ^a^*

Note: Values represent the mean of triplicates ± SE. Values with different letters in a column indicate significant differences (ANOVA, *p* ˂ 0.05) among experimental variants. Values with different symbols */** in a column differ significantly after the challenge test (T-dependent, *p* ˂ 0.05). RBC—red blood cell (erythrocyte) count; Hb—hemoglobin; Ht—hematocrit; MCV—mean corpuscular volume; MCH—mean corpuscular hemoglobin; MCHC—mean corpuscular hemoglobin content.

**Table 5:** Values of hematological parameters of *Cyprinus carpio* under normal conditions.

Carp Body Weight (g)	Ht (%)	Hb (g/dL)	RBC (×10^6^/mm^3^)	MCV (μm^3^)	MCH (pg)	MCHC (g/dL)	References
138.3 ± 28.7	29 ± 3	8.63 ± 0.76	1.64 ± 0.14	180.30 ±15.3	52.9 ± 4.7	29 ± 2.0	[62]
200	31.8 ± 5.5	6.94 ± 1.6	1.81 ± 0.2	178.2 ± 31.7	40.2 ± 6.5	21.6 ± 3.3	[63]
61.2 ± 7.3	22–39	3.76–8.76	0.90–2.02	133.7–248.4	36.9–57.8	15–32	[64]
138 ± 28.7	26 ± 3	6.84 ± 0.9	1.05 ± 0.03	248.1 ± 36	65.2 ± 10	26.3 ± 0.13	[65]
297.4 ± 55.6	29.2 ± 2.7	7.43 ± 0.7	1.63 ± 0.13	179.5 ± 13.5	45.8 ± 4.2	25.5 ± 0.78	[66]
41 ± 0.20	42.2 ± 0.5	11.24 ± 0.41	1.80 ± 0.02	234.5 ± 2.0	62.5 ± 1.6	26.67 ± 0.7	[67]
67.5 ± 9.1	30.9 ± 3.5	7.18 ± 0.13	1.40 ± 0.09	217.2 ± 24.2	51.9 ± 8.2	24.1 ± 0.31	[68]
61.9 ± 2.4	42.8 ± 4	9.4 ± 0.8	1.37 ± 0.19	317.9 ± 60.9	-	22.2 ± 3.3	[69]

**Table 6:** Blood metabolic profile parameters of *Cyprinus carpio*.

Parameter	After the Feeding Experiment/Before APAP Challenge Test					
	M	SM	BBR100	BBR200	SM + BBR100	SM + BBR200
TG (mg/dL)	458 ± 81.93 ^c^	370.33 ± 76.44 ^b^	367.00 ± 67.39 ^b^	347.29 ± 45.74 ^b^	343.5 ± 53.78 ^a^	342.6+ ± 48.71 ^a^
CHOL (mg/dL)	268.33 ± 33.07 ^c^	234.00 ± 16.7 ^b^	206.75 ± 9.81 ^a^	225.00 ± 22.99 ^b^	225.66 ± 12.5 ^b^	214.5 ± 20.69 ^a^
LDL (mg/dL)	115.10 + 4.56 ^d^	98.22 ± 11.22 ^c^	53.67 ± 7.55 ^b^	46.67 ± 9.34 ^a^	64.34 ± 8.56 ^b^	54.76 ± 6.55 ^b^
HDL (mg/dL)	44.71 ± 6.32 ^a^	42.73 ± 8.12 ^a^	77.50 ± 9.10 ^b^	89.88 ± 8.45 ^c^	75.43 ± 6.77 ^b^	82.12 ± 7.45 ^b^
ALT (U/L)	10.25 ± 1.25 ^b^	10.00 ± 3.00 ^b^	8.00 ± 1.82 ^a^	8.12 ± 0.63 ^a^	9.33 ± 4.03 ^a^	8.33 ± 1.96 ^a^
AST (U/L)	174 ± 19.71 ^a^	142.67 ± 49.03 ^a^	134.79 ± 8.86 ^a^	188.67 ± 37.11 ^a^	199.5 ± 55.47 ^a^	147.8 ± 22.81 ^a^
ALP (U/L)	186 ± 48.46 ^a^	158.33 ± 27.28 ^a^	144.67 ± 20.71 ^a^	147 ± 54.72 ^a^	185.66 ± 68.19 ^a^	155.25 ± 77.10 ^a^
GGT (U/L)	1.33 ± 0.47 ^a^	1.23 ± 0.75 ^a^	0.92 ± 0.09 ^a^	1.00 ± 0.44 ^a^	1.38 ± 0.33 ^a^	1.45 ± 0.32 ^a^
BILD (mg/dL)	0.18 ± 0.05 ^a^	0.13 ± 0.03 ^a^	0.15 ± 0.01 ^a^	0.18 ± 0.04 ^a^	0.12 ± 0.03 ^a^	0.15 ± 0.02 ^a^
BILT (mg/dL)	0.31 ± 0.06 ^b^	0.20 ± 0.02 ^a^	0.21 ± 0.06 ^a^	0.23 ± 0.09 ^a^	0.19 ± 0.02 ^a^	0.21 ± 0.01 ^a^
**Parameter**	**After APAP Challenge Test**					
	**M**	**SM**	**BBR100**	**BBR200**	**SM + BBR100**	**SM + BBR200**
TG (mg/dL)	141.00 ± 29.81 ^a^**	189.25 ±38.59 ^b^*	186.75 ± 41.55 ^b^*	181.75 ± 38.81 ^b^*	182.25 ± 28.50 ^b^*	163.25 ± 27.73 ^b^*
CHOL (mg/dL)	187.25 ± 32.01 ^a^**	173.25 ± 14.10 ^a^**	196.50 ± 27.19 ^a^*	188.25 ± 24.40 ^a^*	156.00 ± 25.17 ^a^**	170.50 ± 18.77 ^a^*
LDL (mg/dL)	95.10 + 4.56 ^d^**	78.22 ± 11.22 ^c^**	44.67 ± 7.55 ^b^**	39.61 ± 7.24 ^a^*	44.98 ± 7.65 ^b^**	39.16 ± 8.34 ^b^**
HDL (mg/dL)	34.71 ± 6.32 a**	35.73 ± 8.12 ^a^**	68.20 ± 9.10 ^b^*	82.33 ± 4.95 ^c^*	70.43 ± 7.37 ^b^*	77.12 ± 7.45 ^b^*
ALT (U/L)	42.50 ± 8.58 ^b^**	25.50 ± 8.54 ^a^*	26.00 ± 2.58 ^a^*	29.75 ± 12.39 ^a^*	25.30 ± 0.57 ^a^*	29.75 ± 4.71 ^a^*
AST(U/L)	364.25 ± 78.88 ^b^**	218.00 ± 44.21 ^a^*	295.25 ± 88.68 ^a^**	304.50 ± 32.27 ^a^**	242.00 ± 24.12 ^a^*	273.20 ± 79.95 ^a^*
ALP (U/L)	294.50 ± 15.02 ^c^**	183.67 ± 64.01 ^b^*	173.33 ± 49.80 ^a^*	154.00 ± 77.6 ^a^*	199.5 ± 23.01 ^b^*	179.25 ± 78.54 ^b^*
GGT (U/L)	2.90 ± 0.85 ^c^**	1.55 ± 0.42 ^b^*	1.27 ± 0.25 ^a^*	1.15 ± 0.20 ^a^*	1.50 ± 0.57 ^b^*	2.00 ± 1.55 ^b^*
BILD (mg/dL)	0.26 ± 0.04 ^c^**	0.16 ± 0.03 ^a^*	0.22 ± 0.01 ^b^*	0.24 ± 0.05 ^b^*	0.15 ± 0.04 ^a^*	0.20 ± 0.01 ^b^*
BILT (mg/dL)	0.46 ± 0.06 ^c^**	0.27 ± 0.04 ^a^*	0.31 ± 0.04 ^a^*	0.35 ± 0.10 ^b^*	0.25 ± 0.10 ^a^*	0.34 ± 0.09 ^b^*

Note: Values are Mean ± S.E., *n* = 3. Values with different superscripts in a row differ significantly (ANOVA, *p* ˂ 0.05). Values with different symbols */** in a column differ significantly after treatment (T dependent, *p* ˂ 0.05). ALT—alanine amino-transferase; AST—aspartate amino-transferase; ALP—alkaline phosphatase; GGT—gamma glutamyl-transferase; BILD—direct bilirubin concentration; BILT—total bilirubin concentration; HDL—high-density lipoprotein; LDL—low-density lipoprotein; CHOL—cholesterol; TG—triglycerides.

## Data Availability

All the data are available from the first author, and can be delivered if required.
